# Comparison of one- and two-stage basilic vein transposition for arterio-venous fistula formation in haemodialysis patients: preliminary results

**DOI:** 10.5830/CVJA-2013-077

**Published:** 2013-11

**Authors:** Sedat Ozcan, Ali Ümit Yener, Ali Kemal Gür, Dolunay Odabaşi

**Affiliations:** Department of Cardiovascular Surgery, Çanakkale Onsekiz Mart University, Çanakkale, Turkey; Department of Cardiovascular Surgery, Çanakkale Onsekiz Mart University, Çanakkale, Turkey; Department of Cardiovascular Surgery, Yuzuncu Yil University, Van, Turkey; Department of Cardiovascular Surgery, Yuzuncu Yil University, Van, Turkey

**Keywords:** renal dialysis, arterio-venous fistula, basilic vein transposition, complication

## Abstract

**Objective:**

This study aimed to compare the results of one-and two-stage basilic vein transposition (BVT) in haemodialysis patients.

**Methods:**

This was a non-randomised, retrospective study between January 2007 and January 2012 on 96 patients who were diagnosed with end-stage renal failure (ESRF) (54 males, 42 females; mean age 43.6 ± 14 years) and underwent one- or two-stage BVT in our clinic. All patients who were not eligible for a native radio-cephalic or brachio-cephalic arterio-venous fistula (AVF) were scheduled for one- or two-stage BVT after arterial (brachial, radial and ulnar) and venous (basilic and cephalic) Doppler ultrasonography.

Patients were retrospectively divided into two groups: group 1, basilic vein diameter > 3 mm and patients who underwent one-stage BVT; and group 2, basilic vein diameter < 3 mm and patients who underwent two-stage BVT. In group 1, the basilic vein with a single incision was anastomosed to the brachial artery, followed by superficialisation. In group 2, the basilic vein was anastomosed to the brachial artery and they underwent the superficialisation procedure one month postoperatively. Fistula maturation and postoperative complications were assessed.

**Results:**

The mean diameter of the basilic vein was statistically significantly higher in group 1 (3.46 ± 0.2 mm) than in group 2 (2.79 ± 0.1 mm) (*p* < 0.05). In terms of postoperative complications, thrombosis, haemorrhage and haematoma were significantly higher in group 1 (34, 36 and 17%, respectively) than in group 2 (23, 14 and 6%, respectively) (*p* < 0.05). The rate of fistula maturation was significantly lower in group 1 (66%), compared to group 2 (77%) (*p* < 0.05). Time to fistula maturation was significantly shorter in group 1 (mean 41 ± 14 days), compared to group 2 (mean 64 ± 28 days) (*p* < 0.05).

**Conclusion:**

Two-stage BVT was superior to one-stage BVT due to its lower rate of postoperative complications and higher fistula maturation, despite its disadvantage of late fistula use. Although the diameter of the basilic vein was larger in patients who underwent one-stage BVT, we observed that one-stage BVT was disadvantageous in terms of postoperative complications and fistula maturation.

## Abstract

In recent years, the number of patients requiring haemodialysis (HD) has been rapidly increasing globally, including Turkey. Arterio-venous fistula (AVF) is the most frequently used method in patients with end-stage renal failure (ESRF) for HD.^1^

The Kidney Disease Outcome Quality Initiative (KDOQI) recommends autologous radio-cephalic or brachio-cephalic AVF as a primary method of choice in HD patients, and basilic vein transposition (BVT) as a secondary option.^2^,^3^ In 1976, Dagher *et al.*^4^ first described the technique of BVT for HD. In later years, several techniques were used.^5^-^11^ This study aimed to compare the patency and complication rates of AVF formed by one-stage and two-stage BVT.

## Methods

Between January 2007 and January 2012, 96 patients (54 males, mean age 43.6 ± 14 years) who were not eligible for radio-cephalic and brachio-cephalic AVF via native veins and who underwent BVT were included in this retrospective study. Patients were selected according to basilica vein diameter, which was evaluated with vascular Doppler. Group 1 consisted of patients with a basilic vein diameter > 3 mm and who underwent one-stage BVT (47 patients, 28 males; mean age 42.8 ± 14.5 years), and group 2 contained patients with a basilic vein diameter < 3 mm and who underwent two-stage BVT (59 patients, 36 males; mean age 44.5 ± 13.5 years).

In group 1, the incision was performed through the basilic vein located in the medial condyle of the humerus and axillary area. The vein was carried over the fascia by tying the lateral branches during release of the basilic vein, while the *nervus cutaneus medialis* of the forearm was preserved. The basilic vein in the antecubital fossa was anastomosed to the brachial artery end to side, using 6-0 or 7-0 polypropylene continuous sutures. Following evaluation of the presence of thrill, the fascia and other layers were closed, lifting the vein and protecting the nerve. One month was allowed for the anastomosed graft to heal before the possible trauma of HD injection.

In group 2 patients, the incision was made through the basilic vein located in the medial and lateral condyle of the humerus and was it anastomosed to the brachial artery laterally using 6-0 or 7-0 polypropylene continuous suture. The incisions were closed in the anatomical layers, after the presence of thrill was evaluated.

In the next stage at one month, an incision was made through the basilic vein located in the medial condyle of the humerus and the axillary area. The vein was carried over the fascia by tying the lateral branches during the release of the basilic vein, while the *nervus cutaneus medialis* of the forearm was preserved. Following the evaluation of the presence of thrill, the fascia and others were closed in anatomical layers, lifting the vein and protecting the nerve. Patients whose wounds had healed after a month underwent HD.

Postoperative complications of one- and two-stage BVT, including primary and secondary patency rates, thrombosis, haemorrhage, haematoma, infection and venous aneurysm were retrospectively analysed.

## Statistical analysis

Statistical analysis was performed using Windows SPSS 14.0 (SPSS Inc, Chicago, IL, USA). Normally distributed data, which were expressed as mean ± standard deviation, were assessed using the t-test. The Kolmogorov-Smirnov test was used to analyse normal distribution of the numerical data. Categorical data were examined by Fischer’s exact test. The dual logistic regression test was used to assess the effects of clinical parameters such as haematoma or fistula maturation. A *p*-value of < 0.05 was considered statistically significant.

## Results

While 28 (59%) patients were male and 19 (41%) were female in group 1, 36 (61%) were male and 23 (39%) were female in group 2. The mean follow up was 36 months. The means of age, duration of ESRF, number of AVFs, patency duration, co-morbidities and diameter of the basilic vein and brachial artery are shown in Table 1.

**Table 1:** Demographics Of The Patients

*Variables*	*Group 1 one-stage BVT (n = 47)*	*Group 2 two-stage BVT (n = 59)*	p*-value*
Gender (M/F)	M = 28 (59%)	M = 36 (61%)	NS
	F = 19 (41%)	F = 23 (39%)	NS
Mean age (years)	M = 43.1 (± 16)	M = 44.9 (± 14)	NS
	F = 42.5 (± 13)	F = 44.1 (± 13)	NS
ESRF duration (months)	M = 63.1 (± 17)	M = 61.7 (± 20)	NS
	F = 64.5 (± 18)	F = 63.3 (± 21)	NS
Previously opened AVF	M = 5 (± 1.6)	M = 5.2 (± 1.7)	NS
	F = 5.45 (± 1.7)	F =5.0 (± 1.6)	NS
Hypertension	15	14	NS
Diabetes mellitus	9	11	NS
Heart disease	4	3	NS
Peripheral vascular disease	2	3	NS
Smoking	9	11	NS
Mean LDL-C (mmol/l)	157 ± 26	145 ± 21	NS
Mean basilic vein diameter (mm)	3.46 ± 0.2	2.79 ± 0.1	< 0.05
Mean brachial artery diameter (mm)	3.71 ± 1.4	3.63 ± 1.5	NS

BVT: basilic vein transposition, AVF: arteio-venous fistula, NS: non-significant, LDL-C: low-density lipoprotein cholesterol, ESRF: end-stage renal failure, M = male, F = female.

The diameter of the operated basilic vein was significantly higher in group 1 (3.46 ± 0.2 mm), than in group 2 (2.79 ± 0.1 mm) (*p* < 0.05). There was no significant difference in the diameter of the brachial artery between the groups. Bleeding–clotting times of the groups are shown in Table 1 and there was no significant difference.

**Table 2:** Bleeding–Clotting Times Of The Groups

*Variables*	*Group 1 one-stage BVT (n = 47)*	*Group 2 two-stage BVT (n = 59)*	**
PT (sec)	17 ± 4	16 ± 4	NS
APTT (sec)	38 ± 7	41 ± 7	NS
INR	1.3 ± 0.5	1.5 ± 0.7	NS
Platelet count (10^3^/ml)	385 ± 70	367 ± 67	NS
Bleeding time (min)	6.1 ± 1.3	5.7 ± 1.2	NS
Clotting time (min)	7.1 ± 2.3	7.3 ± 2.1	NS
Protein C (%)	89 ± 28	92 ± 31	NS
D-dimer (ng/dl)	275 ± 73	321 ± 67	NS
Fibrinogen (g/l)	3.2 ± 0.7	2.8 ± 0.5	NS

PT: prothrombin time, APTT: active partial thromboplastin time, INR: international normalised ratio.

The ratio of fistula maturation, as well as postoperative mortality and morbidity rates are shown in Table 3. There was no significant difference in mortality rate, whereas a significant difference was found in morbidity between the groups (*p* < 0.05). The rate of fistula maturation was significantly lower in group 1 (66%) compared to group 2 (77%) (*p* < 0.05). The mean time to fistula maturation was 41 ± 14 days in group 1, while it was 64 ± 28 days in group 2, indicating a significant difference between the groups (*p* < 0.05).

**Table 3:** Complications

*Variables*	*Group 1 one-stage BVT (n = 47)*	*Group 2 two-stage BVT (n = 59)*	p*-value*
Mortality	3 (6%)	2 (4%)	NS
Maturation rate	31 (66%)	45 (77%)	< 0.05
Infection	6 (12%)	5 (10%)	NS
Thrombosis	16 (34%)	11 (23%)	< 0.05
Bleeding	17 (36%)	7 (14%)	< 0.05
Haematoma	8 (17%)	3 (6%)	< 0.05
Pseudo-aneurysm	2 (4%)	3 (6%)	NS
Steal syndrome	4 (8%)	3 (6%)	NS
Oedema	5 (10%)	6 (10%)	NS
Mean fistula maturation time (day)	41 ± 14	64 ± 28	< 0.05
Mean fistula flow rate (ml/min)	280 ± 23	300 ± 31	NS

NS: non-significant.

With regard to auxiliary interventions, the rate of intervention for early (≤ 10 days) fistula thrombosis was significantly higher in group 1 (21%) compared to group 2 (12%). However, there was no significant difference in rate of intervention for late (≥ 10 days) fistula thrombosis between the groups (20% in group 1; 22% in group 2). The number of auxiliary interventions to manage haemorrhage and haematoma following fistula formation was significantly higher in group 1 (17%, 10%) than in group 2 (6%, 2%) (*p* < 0.05). Auxiliary surgical interventions are summarised in Table 4.

**Table 4:** Assisted Interventional Surgery Rates

*Variables*	*Group 1 one-stage BVT (n = 47)*	*Group 2 two-stage BVT (n = 59)*	p*-value*
Early (≤ 10 day) thrombosis	10 (21%)	6 (12%)	< 0.05
Bleeding	8 (17%)	3 (6%)	< 0.05
Haematoma	5 (10%)	1 (2%)	< 0.05
Late (≥ 10 day) thrombosis	9 (20%)	11 (22%)	NS
Pseudo-aneurysm	2 (4%)	3 (6%)	NS
Steal syndrome	2 (4%)	3 (6%)	NS

NS: non-significant.

**Fig. 1.**
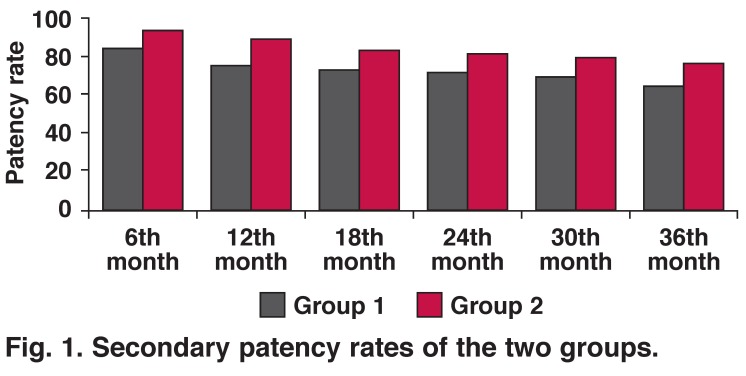
Secondary patency rates of the two groups.

**Fig. 2.**
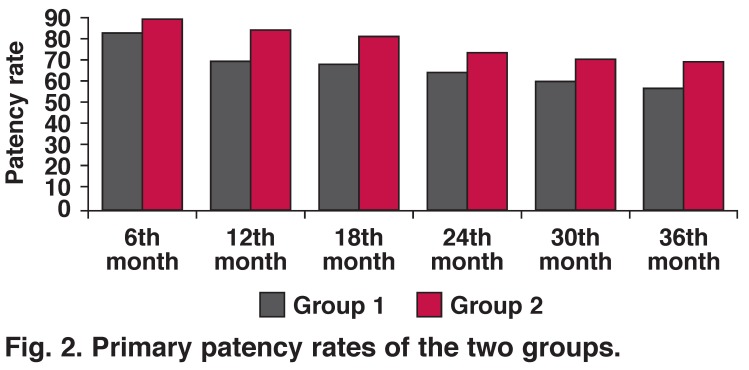
Primary patency rates of the two groups.

Primary and secondary patency rates in both groups are shown in Tables 5, 6, 7, 8. Statistical comparisons of primary/secondary patency rates between the groups are shown in Figs 1 and 2.

**Table 5:** Secondary Patency Rates Of Group 1

*Month*	*n = 47*	*Function loss*	*Function loss rate*	*Patency rate*	*Cumulative patency rate*
6	40	4	0.15	0.85	85.00
12	36	4	0.10	0.90	76.00
18	35	1	0.02	0.98	74.00
24	34	1	0.02	0.98	72.00
30	33	1	0.02	0.98	70.00
36	31	2	0.06	0.94	66.00

**Table 6:** Primary Patency Rates Of Group 1

*Month*	*n = 47*	*Function loss*	*Function loss rate*	*Patency rate*	*Cumulative patency rate*
6	39	8	0.17	0.83	83.00
12	33	6	0.15	0.85	70.00
18	32	1	0.03	0.97	68.00
24	30	2	0.06	0.94	64.00
30	28	2	0.07	0.93	60.00
36	27	1	0.03	0.97	57.00

**Table 7:** Secondary Patency Rates Of Group 2

*Month*	*n = 59*	*Function loss*	*Function loss rate*	*Patency rate*	*Cumulative patency rate*
6	46	3	0.06	0.94	94.00
12	44	2	0.04	0.96	90.00
18	41	3	0.07	0.93	84.00
24	40	1	0.02	0.98	82.00
30	39	1	0.02	0.98	80.00
36	38	1	0.02	0.98	77.00

**Table 8:** Primary Patency Rates Of Group 2

*Month*	*n = 59*	*Function loss*	*Function loss rate*	*Patency rate*	*Cumulative patency rate*
6	43	6	0.12	0.88	88.00
12	41	2	0.04	0.96	84.00
18	39	2	0.05	0.95	80.00
24	36	3	0.07	0.93	73.00
30	35	1	0.03	0.97	71.00
36	34	1	0.02	0.98	69.00

## Discussion

Patients with ESRF must receive HD to survive, until they undergo renal transplantation. AVF surgery to supply extracorporeal blood flow has been performed for many years during HD.^12^ The optimal flow rate is ≥ 200 ml/min with an easy-to-use device, providing sufficient supply in a durable and safe procedure.^13^,^14^ For this purpose, arteries and veins of the upper limbs are mostly used. Alternative methods can be applied for patients without suitable veins.^15^,^16^

In compliance with the KDOQI recommendations, BVT is the most preferred method for fistula formation in our clinic when autologous veins are not suitable to construct radio-cephalic and brachio-cephalic AVF.^2^,^3^ In our study, fistulae formed by one- and two-stage BVT were examined in terms of patency and complication rates.

No significant difference was found between the groups in terms of age, gender, ESRF and the number of fistulae previously formed. In addition, there was no significant difference in co-morbidity or the mean diameter of the brachial artery. The diameter of the basilic vein was significantly larger in group 1 (3.46 ± 0.2 mm) compared to group 2 (2.79 ± 0.1 mm) (*p* < 0.05). There was no significant difference in mortality rate between the groups (6% in group 1; 4% in group 2) or mean flow rate of BVT. Time to fistula maturation was significantly shorter in group 1 (mean 41 ± 14 days) compared to group 2 (mean 64 ± 28 days) (*p* < 0.05).

The rates of postoperative complications, including infection (12% in group 1; 10% in group 2), pseudoaneurysm (4% in group 1; 6% in group 2), steal syndrome (8% in group 1; 6% in group 2), and oedema (10% in group 1; 10% in group 2) were similar, indicating no significant difference between the groups. However, there was a significant difference between the groups in respect of thrombosis (34% in group 1; 23% in group 2), haemorrhage (36% in group 1; 14% in group 2) and haematoma (17% in group 1; 6% in group 2) (*p* < 0.05).

A review of the literature revealed that infection rate was 7% in a study conducted by Dilege *et al.*^17^ and 14% in a study carried out by Veeramanive *et al.*^18^ In our study, the infection rate was 12 and 13% in groups 1 and 2, respectively.

Rivers *et al.*^19^ found the rate of pseudoaneurysm to be 3%. In our study, the rate of pseudoaneurysm was 4 and 5% in group 1 and group 2, respectively.

The rate of steal syndrome was 3.2–6.5% in published studies.^21^-^23^ We found that 8% of the patients in group 1 and 11% of those in group 2 had steal syndrome, indicating a higher rate compared to the literature. A total of 4% of the patients in group 1 and 6% of those in group 2 underwent secondary corrective surgery due to steal syndrome, which is a limb-threatening disease. The incidence of corrective surgery due to steal syndrome was up to 6.5% in the literature.^22^,^24^,^25^ Our results for surgery due to steal syndrome were consistent with that in the literature.

In our study, the rate of fistula maturation was 66% in group 1 and 77% in group 2, indicating a higher rate in group 2, whereas the rate of thrombosis was 34% in group 1 and 23% in group 2, indicating a higher rate in group 1 (*p* < 0.05). Review of the literature revealed that the rate of fistula maturation following BVT was 62–97%.^24^,^26^-^29^

In our study, the mean diameter of the operated basilic vein was significantly higher in group 1 (3.46 ± 0.2 mm) than in group 2 (2.79 ± 0.1 mm) (*p* < 0.05). However, the rate of fistula maturation was higher in group 2, suggesting that the basilic vein that was arterialised using two-stage BVT may have adopted the changes seen in the venous configuration, although this is a controversial issue in the literature.

The rate of patency at 36 months reported by Cantelmo *et al.*^30^ was 57%, while it was 52% at 30 months as reported by Rivers *et al.*^19^ In the literature, the rate of thrombosis was 3–38% with a wide range.^23^,^24^,^26^-^29^

There are few studies in the literature comparing different techniques for BVT.^5^,^8^,^31^ Kakkos *et al.*^31^ compared one-stage and modified two-stage BVT and found that fistula maturation was 85.5% in group 1 and 81.6% in group 2. The authors concluded that there was no significant difference between the groups. In our study, the rate of fistula maturation was higher in group 2 than in group 1, although the mean diameter of the basilic vein was larger in group 1. This is the most important aspect of our study.

The mean diameter of the basilic vein that underwent BVT was not predetermined and it is well known that many factors influence fistula maturation.^1^,^24^,^26^,^28^,^29^,^32^,^33^ In addition, the most important limitation of our study compared to that of Kakkos *et al.*^31^ was the non-randomised design.

With the study limitations, we discuss the possible effects of two complications, haemorrhage and haematoma, on thrombosis and fistula maturation. In our study, a significant difference was observed in terms of haemorrhage (36% in group 1; 17% in group 2) and haematoma (14% in group 1; 6% in group 2) between the groups (*p* < 0.05). Considering an equivalent heparin dose was administered to both groups, the higher rate of haemorrhage and haematoma may have resulted from wider surgical incisions in group 1. However, randomised clinical studies are required to draw a firm conclusion.

Review of the literature revealed that the rate of haematoma was 3.6–11% in other studies.^10^,^11^,^34^ In our study, we found the rate of haematoma to be higher in group 1(17%) than in group 2 (8%). The rate of haematoma in group 2 was therefore consistent with the literature.

With regard to possible factors affecting fistula maturation following BVT, postoperative haematoma and venous hypertension may be more important than the diameter of the basilic vein. This finding is also consistent with data published in the literature.^21^-^23^,^24^,^25^,^31^

With regard to auxiliary interventions, the rate of intervention for early (≤ 10 days) fistula thrombosis was significantly higher in group 1 (21%) than in group 2 (12%). The number of surgeries due to haemorrhage and haematoma was 17 and 10%, respectively in group 1, and 6 and 2%, respectively in group 2 (*p* < 0.05). These findings support the assumption that haemorrhage and haematoma are the most important factors in fistula maturation and thrombosis. There was no statistically significant difference in auxiliary interventions due to late (≥ 10 days) fistula thrombosis (20, 22%), pseudo-aneurysm (4, 6%) and steal syndrome (4, 6%) between the groups.

## Conclusion

AVF formation using BVT is a compelling procedure for the surgeon in order to avoid possible complications, including loss of function, infection, distal ischaemia and venous oedema. Two-stage BVT is superior to one-stage BVT due to its lower rate of postoperative complications, despite the disadvantage of late fistula use. Although the diameter of the basilic vein was higher in our patients who underwent one-stage BVT, we found one-stage BVT was disadvantageous in terms of postoperative complications and fistula maturation. However, we believe the method to be applied should be individually designed until further studies can be performed to establish the superiority of either of these techniques.
